# Early detection of molecular signatures in amyloid conversion and cognitive decline through unbiased plasma proteomics

**DOI:** 10.1002/alz70856_100311

**Published:** 2025-12-24

**Authors:** Jan Muntel, Aida Kamalian, Polina Shichkova, Nicole Barlow, Haley Oller, Jakob Vowinckel, Yuehan Feng, Abhay Moghekar

**Affiliations:** ^1^ Biognosys AG, Schlieren, Zurich, Switzerland; ^2^ Biognosys Inc, Newton, MA, USA; ^3^ Johns Hopkins University School of Medicine, Baltimore, MD, USA

## Abstract

**Background:**

Aging is the greatest risk factor for Alzheimer's disease (AD), yet the biological pathways that distinguish healthy aging from pathological aging, which leads to neurodegeneration, remain poorly understood. There is a critical need for novel biomarkers that can detect the earliest changes in the disease process, particularly well before the onset of cognitive symptoms and preferably even before amyloid conversion that are currently diagnosed by blood/CSF Aβ42/Aβ40 ratio. Plasma represents an ideal biological matrix for novel biomarker discovery due to its ease of collection compared to CSF, its non‐invasive nature, and its suitability for clinical biomarker discovery. To address this challenge, we developed a novel MS‐based proteomics workflow to enable unbiased biomarker discovery.

**Method:**

A sub‐cohort of participants was selected from the BIOCARD cohort. Fifty‐five participants were classified as amyloid converters based on the following criteria: (1) at least three measurements of the CSF Aβ42/Aβ40 ratio spanning over 10 years and (2) two or more early time points with a ratio >0.068 followed by later measurements <0.068. Fifty‐five non‐converters were selected as age‐, sex‐, and interval‐matched controls, with all CSF Aβ42/Aβ40 ratios remaining >0.068.

A total of 578 plasma samples from 110 participants were analyzed using Biognosys’ P2 workflow. Samples underwent P2 particle‐based pre‐treatment, enzymatic digestion to peptides, and subsequent quantitative mass spectrometry analysis. Additional data, including cognitive assessments, clinical biomarker panels, and MRI scans, were collected at each plasma/CSF sampling time point.

**Result:**

Mass spectrometry‐based proteomics provided valuable insights into protein‐ and peptide‐level changes associated with cognitive decline, illuminating the biological pathways involved in the transition from healthy aging to mild cognitive impairment (MCI). Key pathways identified include lipid metabolism, extracellular matrix remodeling, axonogenesis, and synaptic activity.

Integrating proteomics data with available clinical biomarker and cognitive data enhances the ability to pinpoint specific molecular changes associated with cognitive aging. This approach enables the identification of plasma‐based signatures that precede amyloid conversion and cognitive decline, highlighting markers detectable at early time points before participants clinically convert.

**Conclusion:**

This integrated approach bridges the gap between molecular changes and clinical phenotypes, highlighting plasma‐derived biomarkers as a less invasive alternative to CSF collection.

